# Finite Element Study of the Mechanical Response in Spinal Cord during the Thoracolumbar Burst Fracture

**DOI:** 10.1371/journal.pone.0041397

**Published:** 2012-09-24

**Authors:** Ya-Bo Yan, Wei Qi, Zi-Xiang Wu, Tian-Xia Qiu, Ee-Chon Teo, Wei Lei

**Affiliations:** 1 Department of Orthopaedics, Xijing Hospital, Fourth Military Medical University, Xi'an, China; 2 School of Mechanical and Aerospace Engineering, Nanyang Technological University, Singapore; 3 Surgery Department of 520th Hospital of PLA, Mian yang, China; University of Colorado School of Medicine, United States of America

## Abstract

**Background:**

The mechanical response of the spinal cord during burst fracture was seldom quantitatively addressed and only few studies look into the internal strain of the white and grey matters within the spinal cord during thoracolumbar burst fracture (TLBF). The aim of the study is to investigate the mechanical response of the spinal cord during TLBF and correlate the percent canal compromise (PCC) with the strain in the spinal cord.

**Methodology/Principal Findings:**

A three-dimensional (3D) finite element (FE) model of human T12-L1 spinal cord with visco-elastic property was generated based on the transverse sections images of spinal cord, and the model was validated against published literatures under static uniaxial tension and compression. With the validated model, a TLBF simulation was performed to compute the mechanical strain in the spinal cord with the PCC. Linear regressions between PCC and strain in the spinal cord show that at the initial stage, with the PCC at 20%, and 45%, the corresponding mechanical strains in ventral grey, dorsal grey, ventral white, dorsal white matters were 0.06, 0.04, 0.12, 0.06, and increased to 0.14, 0.12, 0.23, and 0.13, respectively. At the recoiled stage, when the PCC was decreased from 45% to 20%, the corresponding strains were reduced to 0.03, 0.02, 0.04 and 0.03. The strain was correlated well with PCC.

**Conclusions/Significance:**

The simulation shows that the strain in the spinal cord correlated well with the PCC, and the mechanical strains in the ventral regions are higher than those in the dorsal regions of spinal cord tissue during burst fracture, suggesting that the ventral regions of the spinal cord may susceptible to injury than the dorsal regions.

## Introduction

The spinal cord is of clinical interest due to its vulnerable high injury severity in burst fracture [1]. The thoracolumbar burst fracture (TLBF) which accounts for about 15% of all spinal fractures is a relatively common cause of spinal cord injury (SCI) in the younger population [1]. During burst fracture, the relationship between the percent canal compromise (PCC) and the mechanical response of spinal cord is still unknown, and may be useful in assessing the neurologic deficits clinically [2]–[3].

Many studies were carried out to investigate the relationship between neurologic deficits and PCC in TLBF [4]–[6]. Some authors considered PCC an important factor in neurological deficit [7]–[8], while others found no such relationship [5]. Panjabi et al. [9] and Wilcox et al. [10] suggested that the final bone fragment position did not represent the maximum dynamic canal occlusion based on the dynamic measurement of the fragment's retropulsion in their experiments. Therefore, some patients with mild canal stenosis have suffered from severe neurological dysfunction. To date, PCC is still an important indicator for assessing the injury severity of spinal cord in burst fracture due to its clinical visibility from radiographic measurements [7]–[8], [11]. Some studies showed that burst fracture with PCC of more than 40% require surgery [12]–[13]. In a retrospective study [11], the computer tomography images of 50 patients who had been surgically treated for a TLBF showed that the PCC was about 42%.

Most published experimental studies on burst fracture [10], [14] mainly focused on the measurement of the PCC caused by the retropulsion of the bony fragment. The mechanical response of the spinal cord during burst fracture was seldom quantitatively addressed. The internal stress or strain within spinal cord, which may be crucial for investigation of the SCI, is not feasible *in vitro* or *in vivo* measurement in human. Many finite element (FE) models as surrogate experiments were simulated to investigate the internal changes of stress and strain distribution within the spinal cord. In literature, the injury mechanism of cervical and thoracic spinal cord has been studied using FE models [15]–[17]. Greaves et al. [15] constructed a FE model of the cervical spine with spinal cord and studied the transverse contusion and distraction injury to spinal cord. Li et al. [16] studied the acute spinal cord injury by internal stress inside the spinal cord under hyperextension using a validated cervical cord model. Sparray et al. [17] demonstrated that the stress and strain of human thoracic spinal cord under compression were affected by changes of tissue resulting from aging and disease. Though, in thoracolumbar spine, some researchers [18]–[20] attempted to conduct the FE studies of the burst fractures, few of these studies included the spinal cord in their FE models. Wilcox et al.'s study [19] developed a bovine thoracolumbar model with spinal cord and investigated the recoil effect to the bony fragment, but, the mechanical response of spinal cord was not documented during the burst fracture simulation. Furthermore, Bain et al. [2] performed an *in vivo* experiment in guinea pigs to determine the quantitative relationship between applied displacement and the resulting strain in the pre-chiasmatic region of the optic nerve. They reported that the tissue Lagrangian strain was a good predictor of *in vivo* axonal injury, suggesting that the morphological injury to neural tissue and the function impairment was associated with the Lagrangian strain.

To the best of our knowledge, few studies look into the internal strain of the white and grey matters within the spinal cord during TLBF, and correlate the corresponding PCC and strain in the spinal cord. Accordingly, in the present study, a 3D FE model of spinal cord was generated to document the computed PCC and internal strains of the white and grey matters of spinal cord in TLBF process.

## Materials and Methods

### FE modeling

**Figure 1:**
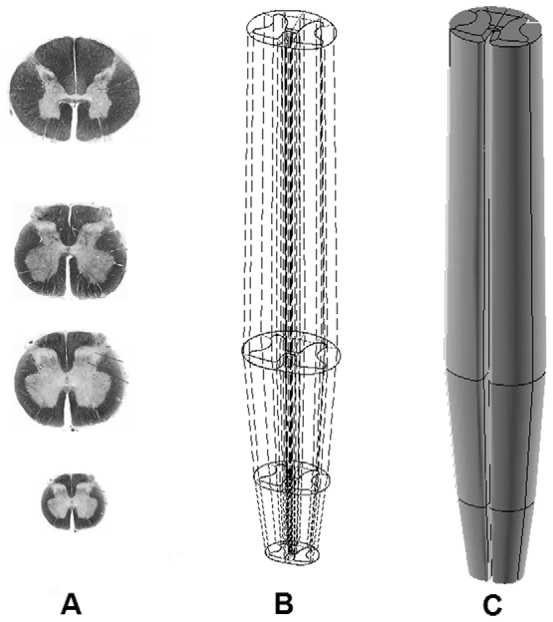
Geometry of thoracolumbar spinal cord. A: Four stained cross sections of spinal cord (From Gray's anatomy for student, 2^nd^ ed., Churchill Livingstone/Elsevier). B: The four outlines were connected with straight lines; C: Full volume of the spinal cord at T-12 and L-1 level.

**Figure 2:**
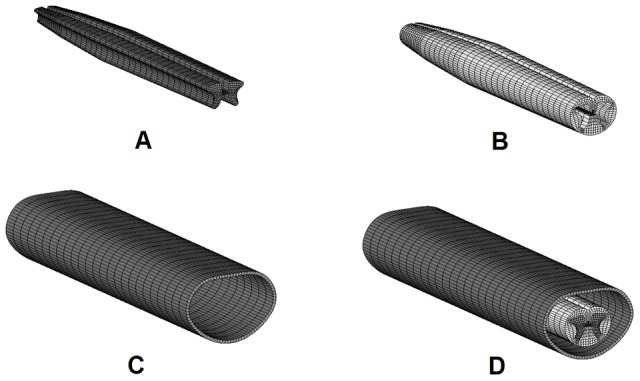
FE model of thoracolumbar spinal cord. A: Grey matter. B: White matter with pia matter; C: Dura matter; D: The assembled spinal cord model.

The FE model of the spinal cord was created based on the cross section images, anatomic data of published anatomic text [21] and quantitative study [22] of spinal cord at T12-L1 region. According to the smooth geometry of the spinal cord [23], four sections were used to reconstruct the geometry of the spinal cord (Fig. 1 A). The images were then imported to ScanIP V4.2 software (SimpleWare, Ltd, Exeter, UK) to process the images to obtain the outlines of the white matter and grey matter from each cross-sectional image of the spinal cord (Fig. 1 B). The scale of the cross-sectional 2D image was adjusted to the actual value based on a previous study [22]. During the scale procedure, the aspect ratio of the image was maintained. Then, the outlines of the grey/white matters were imported to ANSYS/LS-Dyna 11.0 software (ANSYS Inc., Canonsburg, PA, USA) to create areas for volume rendering (Fig. 1 C). The spacing between the adjacent sections was based on the length of the spinal cord in the corresponding spine level [22]. The total length of thoracolumbar spine cord was set as 55 mm according to the statistic data [22]. The spinal cord was assumed to be symmetrical about the mid-sagittal plane, and only half of the spinal cord was reconstructed and the whole model was integrated by reflection. The geometry of the dura with a thickness of 0.4 mm followed the inner contour of spine canal of thoracolumbar spine and the cross section area of the spinal canal adopted in the current study fell in the range of the statistical results reported [24]–[25]. The white, grey matters and dura in the model were then all meshed with eight-node brick element, as shown in Fig. 2 A, B, C, D. A layer of shell element with thickness of 0.1 mm was generated on the outer surface of white matter to represent the structure of pia based on the experimental study [26].

The FE model of the spinal cord structure in present study consisted of grey matter, white matter, pia and dura. Visco-elastic material properties were assigned to all component of spinal cord tissue, except the dura, based on the Bilston et al. study [27]. Quasi-linear viscoelastic material model was used to represent the material properties of the spinal cord (See Appendix S1). The elastic material was assigned to the dura based on Wilcox et al. study [28]. Table 1 and 2 show the material properties adopted in the current study for each part of the spinal cord. For each simulation, contact elements were created for contacting groups of elements of the bony block, dura mater, and spinal cord. A normal stiffness was approximated for these elements to account for the effect of the connective tissue (arachnoid membrane and fat) and cerebrospinal fluid (CSF) in the spaces between the dura and the pia [15].

**Table 1:** Element types and material type of the components of spinal cord model used in the current study.

	Element type	Material type	Density(g/mm^3^)	*E (MPa)*	ν	References
Gray mater	Solid brick	Visco-elastic	1.04e-3	_	_	Bilston et al. 1996 [28];
White mater	Solid brick	Visco-elastic	1.04e-3	_	_	Bilston et al. 1996 [28];
Pia mater	Shell	Visco-elastic	1.13e-3	_	_	Bilston et al. 1996 [28];
Dura mater	Solid brick	Elastic	1.13e-3	142	0.45	Wilcox et al. 2001 [29];

**Table 2:** Material properties of the components of spinal cord model.

Parameters used for the visco-elastic material model									
Parameters	C1	C2	C3	G1	G2	G3	β1	β2	β3
Values	0.5345	1.0665	1.0113	0.8927	0.8926	0.8917	−0.0137	0.0775	0.0357

### Model validation

To validate the model, the FE model of spinal cord, without dura mater, was loaded under uniaxial tension and posterior compression and compared the computed stress-relaxation curve in uniaxial and force-displacement curve with published experimental and simulation results [15], [27], [29]–[31]. In these experimental and simulation studies, the dura mater of the specimen was removed to study the mechanical characteristics of white and grey matters.

**Figure 3:**
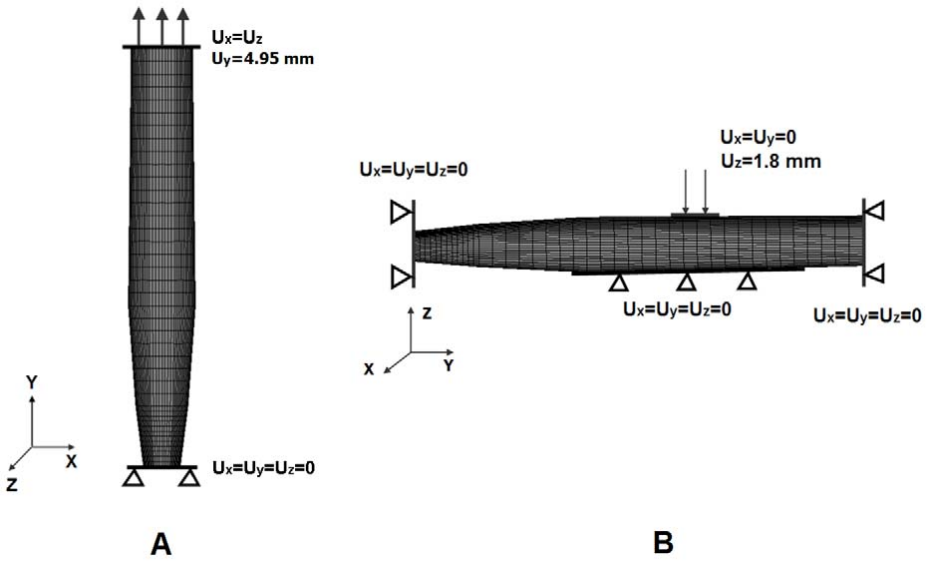
Boundary conditions and loadings. A:Uniaxial tensile simulation; B: Posterior compression simulation.

**Figure 4:**
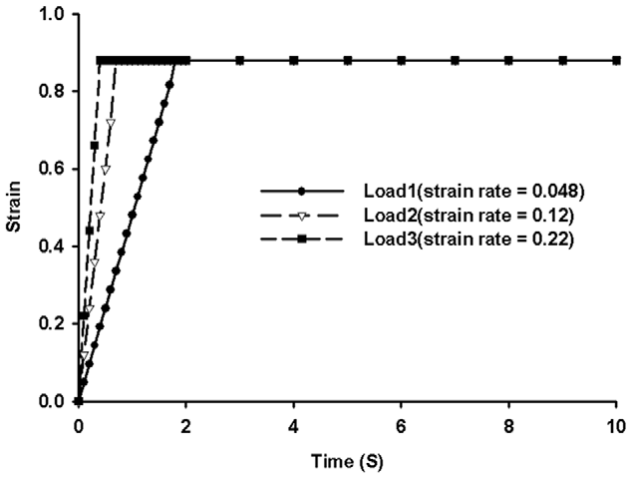
Loading curves for tensile simulation with different strain rates.

In uniaxial tension simulation, the boundary and loading conditions in the FE model were adopted from an *in vitro* experiment [27]. All the nodes at the caudal of the spinal cord model were fully constrained in all directions; an axial displacement of 4.95 mm applied in 10 time steps at strain rates of 0.048, 0.120 and 0.225 sec^−1^ was assigned to all nodes on the top surface (Fig. 3 A). At maximum displacement of 4.95 mm (equivalent to a strain of 9%), the displacement was held constant at this level for 10 seconds. Figure 4 shows the loading curve for spinal cord in uniaxial tensile simulation. The axial reaction forces at the constrained (caudal) end for each time step were extracted to calculate the stress. The corresponding tensile strain for each time step was calculated based on the distractive displacement, and the relationship between stress and strain was plotted for comparison with published experimental results [27], [31].

In posterior compression simulation, both caudal and cephalic ends of the spinal cord were fixed in all directions (Fig. 3 B). The ventral surface of the spinal cord was fixed to simulate the posterior wall of vertebral bodies. A transverse displacement of 1.8 mm in 10 substeps was applied on the dorsal surface of the middle third of the spinal cord with a flat contact area of 25 mm^2^ [30] to simulate the compressive loading according to the experimental study. The compressive reaction forces of the spinal cord for each substep in the dorsal-ventral direction were documented. Then, the curve of reaction force with displacement was plotted and compared with the data of the published *in vivo* static compression study of the spinal cord [15], [29]–[30].

### Impact simulation

For simulation of the impact load on the FE model of spinal cord with dura mater, a rectangular block with dimension of 12×12×20-mm^3^ was used to represent the bony fragment impacting the spinal cord with the surface of 12×12 mm^2^ [14]–[15]. As the actual fragments have a cortical surface and a large cancellous component, the material properties of surface with thickness of 1 mm contacting the spinal cord was assigned as cortical bone (with a Possion's ratio of 0.3 and an elastic modulus of 12 GPa) and the material properties of other part of the block was assigned as cancellous bone (with a Possion's ratio of 0.3 and an elastic modulus of 0.1 GPa).

**Figure 5:**
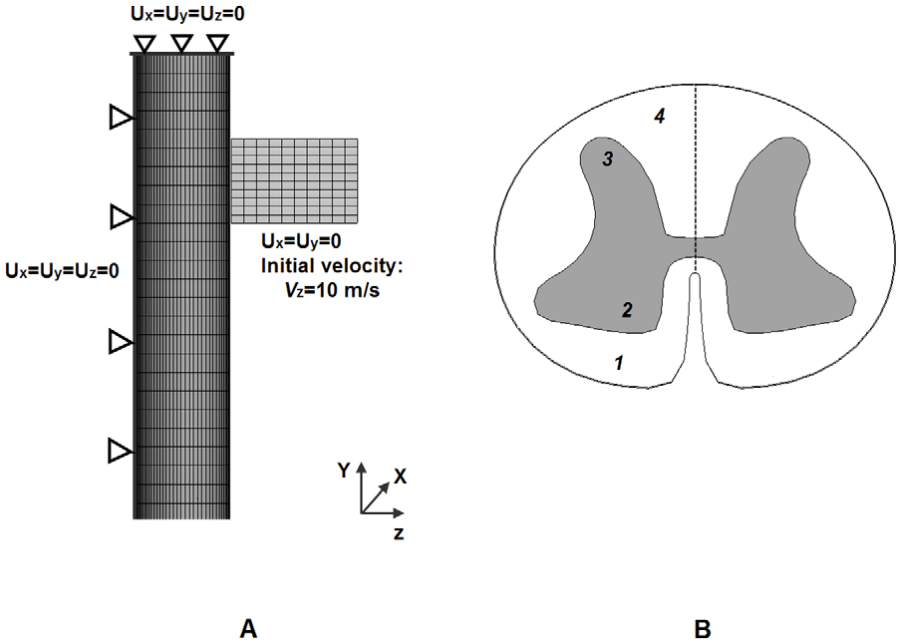
Simulation and regions in spinal cord tissue. A: Boundary conditions and loading of burst fracture simulation; B: Four regions in spinal cord tissue. 1: Ventral region of white matter, 2: Ventral region of grey matter, 3: Dorsal region of grey matter, 4: Dorsal region of white matter.


To represent the human situation, the cephalic end of the spinal cord with dura was fully fixed in all directions while the caudal end of the spinal cord with dura was free in all directions. The nodes of the dorsal dura were fixed to simulate the posterior wall (lamina) of spinal canal. The bony block was placed with a small gap of 0.1 mm to the ventral dura surface. An initial velocity of 10 m/s [10] was assigned to the bony block (Fig. 5 A) to simulate impact to the spinal cord by the extruded fragment into the spinal canal during burst fracture. To observe the recoil of the bony block, the total time for the impact simulation was set as 45 ms. The reaction forces were extracted and von Mises strain distributions in four regions (two regions in grey matter and two regions in white regions as shown in Fig. 5 B) of spinal cord tissue in transverse section were plotted. The cross sections at the level of injury of the spinal cord in different time were extracted for calculation of the PCC according to the formula:
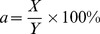
Where:

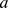
 = PCC;


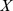
 = The area occupied by the bony block in transverse cross section.


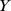
 = Mean of the spinal area of the spinal canal one segment above and below the level of injury (values from literatures [24]–[25]).

The curve between the PCC and time was plotted to evaluate the procedure of impact of bony block and recoil of spinal cord tissue. The curves of strains in four regions of spinal cord with PCC were plotted to assess the injury mechanism of the spinal cord tissue during burst fracture.

In burst fracture, the impact procedure consists of two phases: the impact phase and recoil phase [10]. In the recoil phase, the PCC appear to be smaller than that at the impact phase, and it is hypothesized that spinal cord injury at maximum PCC. It is significant to identify the link between the PCC and the strain in spinal cord at impact phase. Therefore, linear regression was performed to explore the relationship between strain in spinal cord and PCC at impact phase.

## Results

### Validation

**Figure 6:**
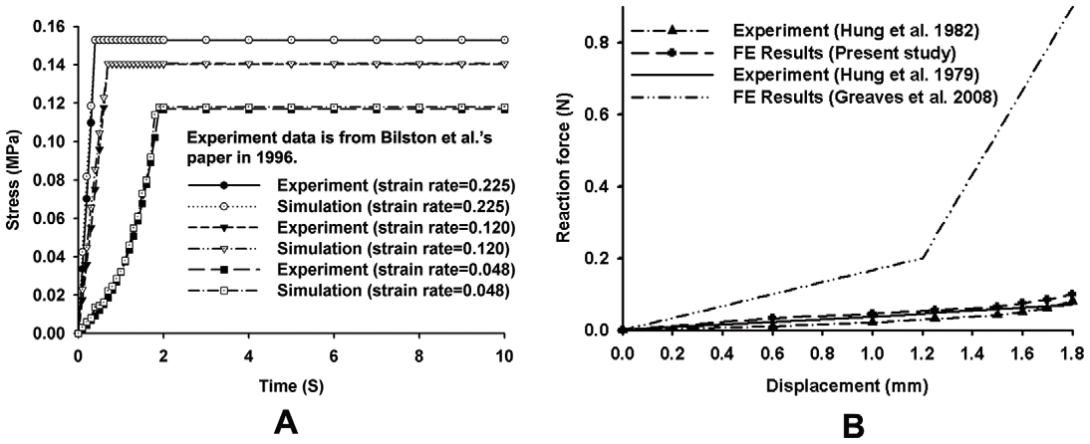
Comparison of present study and experiments. A: Comparison of stress-strain curves of present study and experiment in uniaxial tension; B: Comparison of force-displacement curves under compression between present study prediction and published results.

Figure 6A shows good agreement in the stress-relaxation curve of the spinal cord without dura under static distraction loading based on current FE analysis and the experimental studies [27] under different strain rates. For validation under compression simulation, the computed force-displacement response of the spinal cord from FE analysis against those of the experimental study performed by Hung et al. [30] and a FE model of by Greaves et al. [15] was showed in Fig. 6B. The present study showed non-linear reaction force-displacement curve similar to the experimental results reported by Hung et al. [30] against linear FE results obtained by Hung et al. [30], but within the same trend. At maximum compressive displacement of 1.8 mm, the computed reaction force of present study of 0.1 N fall within the range of the published results of 0.08–0.9 N [15], [30].

### Impact simulation: PCC vs. time

**Figure 7:**
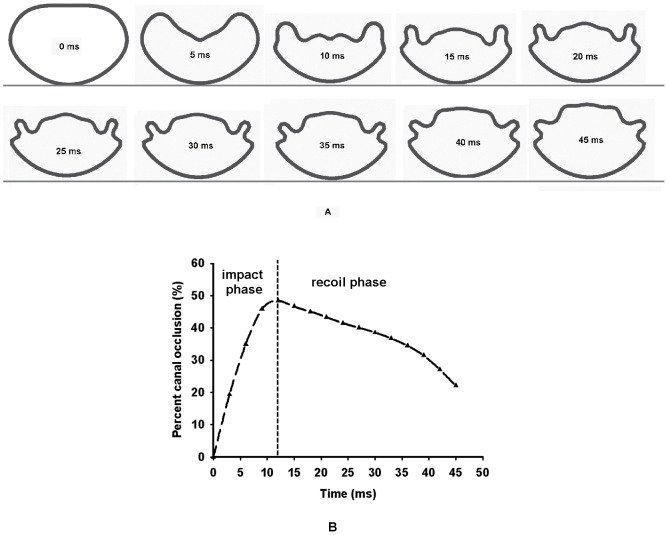
Change of cross sections and PCC *VS.* time. A: cross sections of dura at the injury level during the burst fracture simulation; B: Curve of PCC *VS.* time.

Fig. 7 A showed that the changes in the cross section of the dura at the injury level during impact. Firstly, there was a decrease in the cross section area of dura at the onset till 12 ms, then recovered in to its initial sectional areas and shape in the recoiled phase. Figure 7B shows the curve of PCC with time. The curve was divided into two parts:- the impact phase and the recoiled phase, by the vertical line passing through the peak PCC point. It can be seen that the PCC increased with time during impact phase and peak at 45% when the bony fragment reached its maximum position at 12 ms. In the recoil phase, the spinal cord tissue recoiled, the PCC decreased with time and reached 20% at 45 ms.

### Impact simulation:- strain distribution vs. PCC

**Figure 8:**
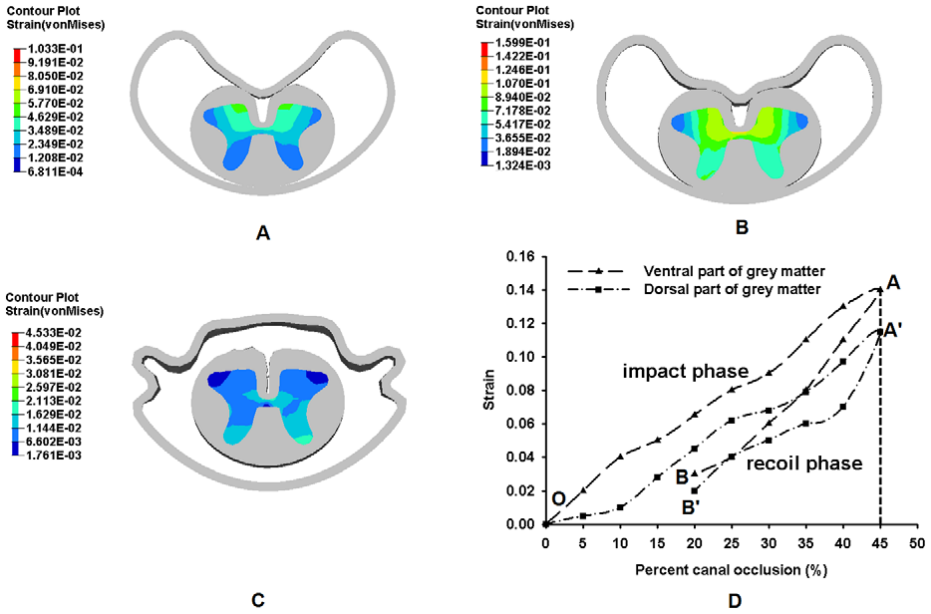
Strain distribution in the grey matter with PCC. A: at 20% (impact phase); B: at 45% (impact phase); C: 20% (recoil phase); D: The curve of maximum von Mises strain in the grey matter with the PCC.

**Figure 9:**
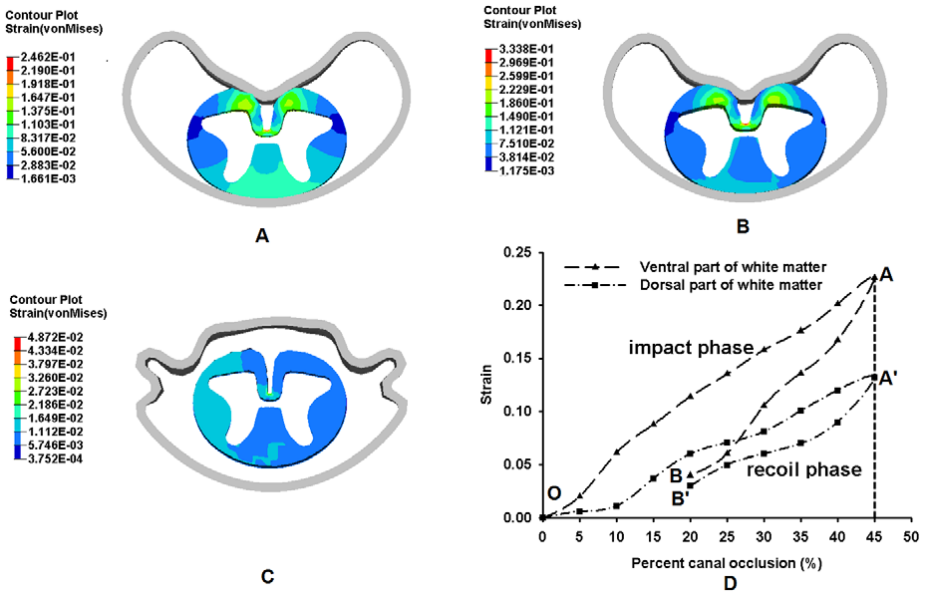
Strain distribution in the white matter with PCC. A: at 20% (impact phase); B: at 45%; C: at 20% (recoil phase); D: The curve of maximum von Mises strain in the white matter with the PCC.

Figures 8 and 9 show the distinct differences in strain distributions between the ventral and dorsal parts of the grey matter and white matter of the spinal cord, respectively, during the burst fracture simulation.

For the grey matter, in Figure 8D, the strain-PCC curve was divided into two phases:- impact phase (OA and OA′) and recoil phase (AB and A′B′) with A and A′ at 45% peak PCC. During the impact phase, the strain in ventral and dorsal regions of grey matter increased with the PCC, and with PCC at 20%, the corresponding strains were 0.06 and 0.04, respectively, as shown in Figure 8A. At 45% PCC, the corresponding strains were further increase to 0.14 and 0.12, respectively (Figure 8B). In the recoil phase, the strains decrease with PCC, and at 20% PCC, the strain in ventral and dorsal regions were 0.03 and 0.02, respectively. (Fig. 8C).

For the white matter, Figure 9A–9D show the strain distribution in white matter increased with the PCC, with higher strain in ventral region than that in dorsal region (Fig. 9A–C). In the impact phase, when the PCC increased to 20% (OA and OA′), the strain in the ventral and dorsal regions were 0.12 and 0.06, respectively (Fig. 9A), and reached maximum values of 0.23 and 0.13, respectively, at 45% PCC (Fig. 9B). In the recoil phase, with the PCC 20% (AB and A′B′), the strains in ventral and dorsal regions of white matter were 0.04 and 0.03, respectively (Fig. 9C).

### Linear regression of strain in spinal cord vs. PCC at the impact phase

**Figure 10:**
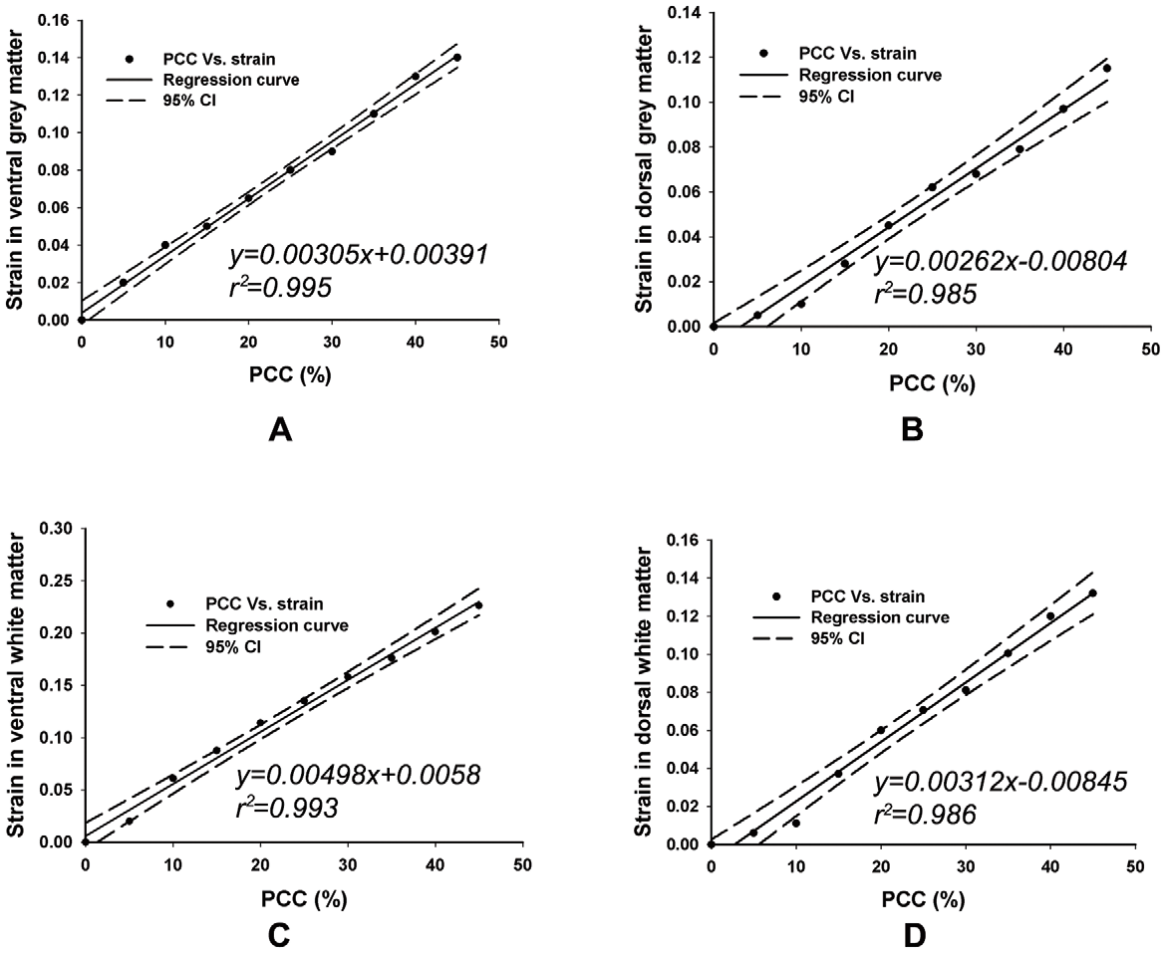
Linear regression of strain Vs. PCC. A: in ventral grey matter; B: in dorsal grey matter; C: in ventral white matter; D: in dorsal white matter..

To explore the relationship between the strain at the spinal cord and the PCC, the computed data were plotted. With best fit curves at 95% prediction bounds, the strains of the grey and white matter in the ventral and dorsal regions were shown in Figure 10. The results showed that the strain in the four parts of the spinal cord cross-sectional areas increase with the PCC.

## Discussion

In this current study, a 3D FE model of the human thoracolumbar spinal cord from T12-L1 level was generated based on published statistical anatomic data [22]–[23] to investigate the injury of spinal cord caused by the bony fragment during thoracolumbar burst fracture. The impact of the fragment on the spinal cord was simulated by the bony block with an initial velocity, and the responses of the spinal cord during the progress of the bony fragment's retropulsion in terms of the PCC and the strain distribution in the cross section of spinal cord were quantitatively documented and correlated.

The development of FE model requires a number of assumptions regarding the geometry, materials, and interactions between components. There are some limitations in this initial spinal cord model.

The components, such as epidural fat and cerebrospinal fluid (CSF), between the posterior wall of vertebral body and the spinal cord that provide cushion and space protecting against mild canal compromise were not modeled. Firstly, it is impractical to measure the volume and shape of epidural fat and CSF; secondly, even in the experimental studies, it is also difficult to reserve the CSF during the specimen preparation [10], [14]. Thirdly, the CSF had the property of fluid which is not computation-efficient to simulate the fluid-solid couple effect of the CSF. Hence, we adopted the methods based on previous studies [15], [18]–[19] using contact elements with stiffness simulating the cushion effect of the epidural tissue and CSF in this study. Accordingly, the cerespinal fluid and arachnoid membrane between the dura and the pia were simulated as contact elements with stiffness to approximate the effect of the connective tissue (arachnoid membrane and fat) and cerebrospinal fluid (CSF) based on the Greaves et al. [15].

Other spinal tissues, such as the posterior longitudinal ligament, dentate ligaments and ligamentum flavum that provide spinal motion stability and cushion effect in the spinal cord during TLBF, were not simulated in the current study. We aim to increase the complexity of such modeling techniques by incorporating these elements into the model in future study.

Due to the limited data available, and with no consensus on the material properties of white and grey mater [32]–[34], in the current study, we adopted similar material properties of the spinal cord based on the Bislton et al. [27] for the FE model, and validated against Bilston et al. results [27].

In the validation study, the stress-relaxation curves and reaction force-displacement curves were non-linear as shown in Fig. 6A and 6B, and were in agreement with published experimental and other FE results published in literature. Though the data used for comparison is from the human cervical and thoracic spinal cord [27], the cat spinal cord [29]–[30] and the rat spinal cord [31] instead of the mechanical response of the thoracolumbar spinal cord, we believe there are some correlations.

During burst fracture simulation, the initial velocity of the bony block was set as 10 m/s based on the Wilcox et al.'s experiment [10]. The computed results showed that the spinal canal area occupied by the bony block in transverse cross section remained constant after 45 ms, and the strain in the spinal cord continued to dissipate with magnitude of strain much lower than that at time of 12 ms (the peak time). Therefore the time of simulation was set to terminate at 45 ms.

As adults' spinal cords usually end at the L1 vertebral body level, its end (known as medullary cone) floats in the cerespinal fluid with a relatively large range of motion [21]. And, in real life, the impact time is very short during the high velocity impact, the boundary condition of the caudal end of the spinal cord does not affect significantly to the results. Therefore, in the study, the boundary condition assigned to the caudal end of the spinal cord i.e. ‘free in all directions’ was reasonably simulated.

Quantifying the strain distribution in different regions of spinal cord tissue with PCC provides insight into the potential effects of spinal cord injury mechanism during TLBF. Furthermore, determination of strain distribution in different regions (white and grey matter) is necessary for understanding the injury of each region and injury classification and pattern of the spinal cord. The results of our study showed that strains are higher in ventral regions of spinal cord during burst fracture (Fig. 8 A–D and Fig. 9 A–D). As there is wider gap between the pia and posterior wall of spinal canal, the white and grey matters may shift posteriorly and the anterior tissues will cushion much of theimpact energy during impact.

Currently, the PCC is a convenient indicator for surgical decision for treatment of spinal cord due to TLBF. It was reported that the magnitude of PCC provided much accurate method for evaluating neural canal encroachment than other methods [11]. Some researchers suggested PCC larger than 40% as a surgical indication for TLBF [12]. From the present study, the strain in the spinal cord increased gradually before 5% of PCC. The strain in the dorsal regions gradually increased with the spinal cord continually being pushed toward the posterior wall of spinal canal. After 10% of PCC, the strains in dorsal regions began to increase significantly (Fig. 8 and Fig. 9D). The gap between the white/grey matters and posterior wall of vertebral body in the current FE model might contribute to the delay of the impact of the bony fragment to some extent. With further increase of PCC, the bony fragment began to compress the spinal cord. Thus, the ventral region of the white matter was compressed first; resulting in strain increasing and propagating.

The linear regression analysis showed that the the strain in the spinal cord correlated well with PCC (Fig. 10A–D). The determination of the relationship within the PCC in radiology, the strain in the spinal cord and the injury severity to the spinal cord would be more helpful and significant to the clinicians. In our future work, we will consider to investigate the relationship between strain and injury severity in the spinal cord in animal test. To our best knowledge, the relationship between strain and injury severity in the neuron studied by Bain et al. [2] showed that axonal injury was produced by dynamically stretching the optic nerve of a adult male guinea pig. Morphological injury was detected with neurofilament immunohistochemical staining. The logistic regression analysis, combined with sensitivity and specificity measures and receiver operating characteristic curves were used to predict strain threshold for axonal injury. The threshold ranged from 0.14 to 0.34, and the optimal threshold strain criterion that balanced the specificity and sensitivity was 0.21.

The variation in strain distribution in different regions with PCC also has implications for clinical understanding of spinal cord trauma. This study indicated that ventral regions of white matter would be injured at the impact phase of the burst fracture (OA and OA′ in Fig. 8D and 9D). When the PCC reached its maximum value (points A and A′ in Fig. 8D and 9D), the injury to the ventral regions could be more severe than that of the dorsal regions. (Fig. 8 A–C and Fig. 9 A–C). The ventral regions plays a role in motor function and the dorsal region is responsible for sensory function. Hence, it could be deduced that the impairment of motor function in ventral regions would be more severe than the sensory function in dorsal region during the burst fracture. Comparison of the motor function and the sensory function impairment of the spinal cord could also provide an explanation about the discrepancy between the radiographic imaging and the neurological deficit [35].

Based on present simulation, we found that the PCC decreased as the bone fragment recoiled, resulting in final PCC was smaller than the maximum value occurred during impact stage. Therefore, the spinal cord might be damaged during the impact when burst fracture occurred in the thoracolumbar spine, and the injury of the spinal cord could not be evaluated by the final canal compromise because of the recoil of the spinal cord. This correlated well with the finding that the final position of the bony fragment probably does not represent the maximum level of the canal occlusion reported by Wilcox et al. [10].

The strain *vs.* PCC curves in Fig. 8D and 9D have two phases corresponding to impact and recoil, respectively, with a turning point at the maximum strain. The impact of bony block used to simulate the fragment in the FE model on the spinal cord caused the strain in the spinal cord to increase to maximum, after which the material elasticity of the spinal cord resulted in recoiling of the bony block. Therefore, the damage to the cord tissue might relate to the peak value of the strain cord instead of the strain measured at the end of the simulation. This could provide an explanation to the phenomenon that patients with mild canal compromise had severe neurological deficit in clinic [36]–[38].

The current simulation also implied that the strain distribution (pattern and magnitude) within the white and grey matters was different. The strain magnitude in white matter was higher than that in the grey matter during the impact procedure (Fig. 7D and Fig. 8D) due to their different materials' properties [23], [39]. When the spinal cord tissue was impacted and pushed to contact the posterior wall of spinal canal by the bony block, the white matter was compressed directly, and then the impact was transmitted to grey matter. Therefore, the strain was higher in white matter. Some researcher tried to find the critical strain for injury threshold of neurological system [2], but it was technically difficult to find the injury threshold in spinal cord. Therefore, it is hard to evaluate the injury severity in the spinal cord using the strain distribution and magnitude. The injury thresholds of white and grey matters need to be further studied.

This study demonstrated that spinal cord might be compressed severely during the impact when burst fracture occurred in the thoracolumbar spine, and the injury to the spinal cord could not be evaluated by the final PCC because of the recoil of the bony block. The ventral parts of the spinal cord are susceptible to the risks of injury compared to those of dorsal parts. This may imply that the impairment to the motor function is more severe than that to the sensory function during burst fracture.

## Supporting Information

Appendix S1 (DOC)
