# Correction: Finite Element Study of the Mechanical Response in Spinal Cord during the Thoracolumbar Burst Fracture

**DOI:** 10.1371/annotation/9975dca7-420a-425e-a513-b645b120edf8

**Published:** 2012-11-30

**Authors:** Ya-Bo Yan, Wei Qi, Zi-Xiang Wu, Tian-Xia Qiu, Ee-Chon Teo, Wei Lei

Lie-Cun Gao was not included in the author byline. Please view the correct author order, affiliations, and citation here:

Wei Qi**^1,4#^**, Liu-Cun Gao**^2#^**, Ya-Bo Yan**^1,3#^**, Zi-Xiang Wu**^1^**, Tian-Xia Qiu**^3^**, Ee-Chon Teo**^3^**, Wei Lei^1*^

**1** Department of Orthopaedics, Xijing Hospital, Fourth Military Medical University, Xi’an, China, **2** State Key Laboratory of Cancer Biology, Xijing Hospital of Digestive Diseases, Fourth Military Medical University, Xi’an, China, **3** School of Mechanical and Aerospace Engineering, Nanyang Technological University, Singapore, **4** Surgery Department of 520th Hospital of PLA, Mian yang, China

Qi W, Gao L-C, Yan Y-B, Wu Z-X, Qiu T-X, Teo E-C, et al. (2012) Finite Element Study of the Mechanical Response in Spinal Cord during the Thoracolumbar Burst Fracture. PLoS ONE 7(9): e41397. doi:10.1371/journal.pone.0041397

