# Supplementary material for: Finite Element Study of the Mechanical Response in Spinal Cord during the Thoracolumbar Burst Fracture
Source: PLoS One. 2012 Sep 24;7(9):e41397. doi: 10.1371/journal.pone.0041397 (PMC3454413; doi:10.1371/journal.pone.0041397)
Supplement: Appendix S1 — (DOC) [file pone.0041397.s001.doc]

## Appendix:

The analytic form of stress vs. time function was different between Biston’s experiment and that used in the software of ANSYS/LS-Dyna. Therefore, we need to deduce the parameters for simulation based on the Biston’s experiment. The methods is following:

In the bilston’s paper (1996), the analytic form of stress vs. time is:

$\sigma(t)=AB\mathop{\varepsilon_{0}}\limits^{\bullet} \sum_{i=1}^{3} G_{i}e^{-t/\tau_{i}}\left(\frac{e^{B\mathop{\varepsilon_{0}}\limits^{\bullet} t}-e^{-t/\tau_{i}}}{\frac{1}{\tau_{i}}+B\mathop{\varepsilon_{0}}\limits^{\bullet} }\right),0<t<t_{0}$ Eq. 1

$\sigma(t)=AB\mathop{\varepsilon_{0}}\limits^{\bullet} \sum_{i=1}^{3} G_{i}e^{-t/\tau_{i}}\left[\frac{e^{t_{0}(\frac{1}{\tau_{i}}+B\mathop{\varepsilon_{0}}\limits^{\bullet} )}-1}{\frac{1}{\tau_{i}}+B\mathop{\varepsilon_{0}}\limits^{\bullet} }\right],t>t_{0}$ Eq. 2

The parameters in the equations above was:

|  | A | B | G1 | τ1 | G2 | τ2 | G3 | τ3 |
| --- | --- | --- | --- | --- | --- | --- | --- | --- |
| Average | 0.028 | 25.9 | 0.104 | 4.38 | 0.121 | 0.554 | 0.377 | 234.4 |
| SD | 0.016 | 12.6 | 0.056 | 0.99 | 0.070 | 0.219 | 0.144 | 124.0 |

Then, the analytic form of quasi-linear viscoelastic model in Ansys/Ls-Dyna is MAT176. The analytic form is:

$$\sigma(t)=\int_{-\infty}^{t} G(t-\tau)\frac{\partial\sigma^{e}(t)}{\partial\varepsilon}\frac{\partial\varepsilon}{\partial\tau}d\tau$$

Reduced relaxation function: $G(t)=\sum_{i=1}^{3} G_{i}e^{-\beta_{i}t}$

Instantaneous elastic response: $\sigma(\varepsilon)=\sum_{i=1}^{3} C_{i}\varepsilon_{i}$

Stress response:

$$\begin{matrix} \sigma(t)=2C_{2}\mathop{\varepsilon_{0}^{2}}\limits^{\bullet} \sum_{i=1}^{3} G_{i}(\frac{e^{-\beta_{i}t}-1}{\beta_{i}^{2}}+\frac{t}{\beta_{i}})-C_{1}\mathop{\varepsilon_{0}\sum_{i=1}^{3} G_{i}\frac{e^{-\beta_{i}t}-1}{\beta_{i}^{2}}}\limits^{\bullet} \\ +3C_{3}\mathop{\varepsilon_{0}^{3}}\limits^{\bullet} \sum_{i=1}^{3} G_{i}(\frac{\beta_{i}^{3}t^{3}-3\beta_{i}^{2}t^{2}+6\beta_{i}t-6}{\beta_{i}^{4}}+\frac{6}{\beta_{i}^{4}e^{\beta_{i}t}}),0<t<t_{0} \end{matrix}$$

$$\begin{matrix} \sigma(t)=2C_{2}\mathop{\varepsilon_{0}^{2}}\limits^{\bullet} \sum_{i=1}^{3} G_{i}(\frac{\beta_{1}t_{0}e^{\beta_{i}t_{0}}-e^{\beta_{i}t_{0}}+1}{\beta_{i}^{2}e^{\beta_{i}t}})+C_{1}\mathop{\varepsilon_{0}}\limits^{\bullet} \mathop{\sum_{i=1}^{3} G_{i}\frac{e^{\beta_{i}t_{0}}-1}{\beta_{i}e^{\beta_{i}t}}}\limits\\ +3C_{3}\mathop{\varepsilon_{0}^{3}}\limits^{\bullet} \sum_{i=1}^{3} G_{i}[\frac{e^{\beta_{i}t_{0}}(\beta_{i}^{3}t^{3}-3\beta_{i}^{2}t^{2}+6\beta_{i}t-6)}{\beta_{i}^{4}e^{\beta_{i}t}}+\frac{6}{\beta_{i}^{4}e^{\beta_{i}t}}],t>t_{0} \end{matrix}$$

To get the parameters in the stress vs. time equation of MAT176, a method proposed by Abramowitch and woo (2004) was used for fitting QLV constants in which the stress from the ramp up strain and the stress following the ramp up strain are consider as additive parts to the objective function. We have the following objective function:

$$\mathop{Min}\limits_{G_{1},G_{2},G_{3},C_{1},C_{2},C_{3},\beta_{1},\beta_{2},\beta_{3}} \sum_{t_{i}=0}^{t_{0}} [\sigma_{i}^{Bilston}(t_{i})-\sigma_{i}^{Mat176}(t_{i})]^{2}+\sum_{t_{j}=0}^{t^{\infty}} [\sigma_{j}^{Bilston}(t_{j})-\sigma_{j}^{Mat176}(t_{j})]^{2}$$

After the optimization, we get the paramaters for the material model for MAT176 in ANSYS/LS-Dyna (tabulated in table 2).
